# Visual Motion and Form Integration in the Behaving Ferret

**DOI:** 10.1523/ENEURO.0228-19.2019

**Published:** 2019-08-20

**Authors:** Erika Dunn-Weiss, Samuel U. Nummela, Augusto A. Lempel, Jody M. Law, Johanna Ledley, Peter Salvino, Kristina J. Nielsen

**Affiliations:** 1 Solomon H. Snyder Department of Neuroscience, Johns Hopkins University School of Medicine, Baltimore, MD 21205; 2 Zanvyl Krieger Mind/Brain Institute, Johns Hopkins University, Baltimore, MD 21218

**Keywords:** behavior, electrophysiology, ferret, form vision, motion vision, visual cortex

## Abstract

Ferrets have become a standard animal model for the development of early visual stages. Less is known about higher-level vision in ferrets, both during development and in adulthood. Here, as a step towards establishing higher-level vision research in ferrets, we used behavioral experiments to test the motion and form integration capacity of adult ferrets. Motion integration was assessed by training ferrets to discriminate random dot kinematograms (RDK) based on their direction. Task difficulty was varied systematically by changing RDK coherence levels, which allowed the measurement of motion integration thresholds. Form integration was measured analogously by training ferrets to discriminate linear Glass patterns of varying coherence levels based on their orientation. In all experiments, ferrets proved to be good psychophysical subjects that performed tasks reliably. Crucially, the behavioral data showed clear evidence of perceptual motion and form integration. In the monkey, motion and form integration are usually associated with processes occurring in higher-level visual areas. In a second set of experiments, we therefore tested whether PSS, a higher-level motion area in the ferret, could similarly support motion integration behavior in this species. To this end, we measured responses of PSS neurons to RDK of different coherence levels. Indeed, neurometric functions for PSS were in good agreement with the behaviorally derived psychometric functions. In conclusion, our experiments demonstrate that ferrets are well suited for higher-level vision research.

## Significance Statement

The ferret is a central animal model for development because of its early parturition. To date, most visual development research in ferrets has focused exclusively on early visual stages. Here, we use behavioral experiments to demonstrate that adult ferrets are capable of visual motion and form integration. These complex visual functions are usually associated with higher-level visual areas in monkeys and ferrets. We similarly observed good agreement between the motion integration performance of neurons in PSS, a higher-level motion area in the ferret, and the behaviorally measured motion integration capacity. Our experiments in the adult ferret demonstrate that the ferret is a viable model for higher-level vision research, which provides exciting opportunities for developmental research in this species.

## Introduction

Because of their early parturition, ferrets are uniquely suited for developmental research. Indeed, research in ferrets has contributed significantly to our understanding of the development of early visual stages (Sharma and Sur, 2014). In contrast, higher-level visual areas have received little attention in this species, even in adult animals. Behaviorally, ferrets have been shown to be capable of basic object discrimination and motion detection tasks (Doty et al., 1967; Hupfeld et al., 2006). Anatomically, ferrets have a relatively large visual system with ∼19 areas (Homman-Ludiye et al., 2010), suggesting extensive processing of visual information beyond primary visual cortex. Consistent with this notion, we have recently demonstrated that one of these areas, area PSS (Philipp et al., 2006), can be considered a higher-level motion area comparable to primate MT (Lempel and Nielsen, 2019). These findings suggest the feasibility of studying higher-level vision in ferrets, which would significantly enhance our ability to investigate its development. To further establish higher-level vision research in ferrets, we addressed two central aspects here: First, we used behavioral experiments to investigate whether ferrets are able to perceptually integrate motion and form signals, functions that are usually associated with higher-level visual areas (Ungerleider and Pasternak, 2004). Second, we tested whether PSS responses are consistent with the behaviorally observed motion integration.

**Figure 1.**
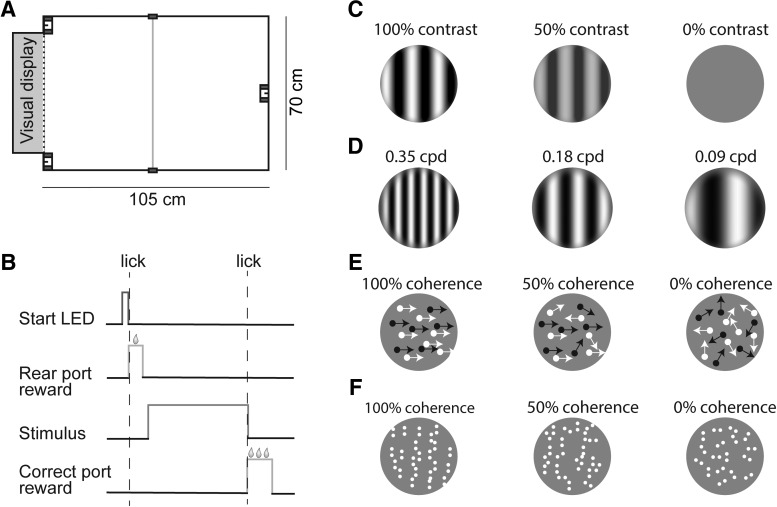
Freely-moving setup and basic stimulus design. ***A***, Schematic of freely-moving behavioral setup. The trial initiation port was centered on the wall opposite the visual display. Choice ports were on either side of the display. An IR beam was also placed across the middle of the box. ***B***, Task structure. An LED was illuminated above the trial initiation port to signal that the ferret could initiate a trial. When the ferret broke the IR beam in that port, a small reward was dispensed, and stimulus presentation was triggered. Subsequent selection of the correct port resulted in ending stimulus presentation and reward delivery. ***C*** & ***D***, Acuity task stimuli: sinusoidal gratings with varying contrasts and spatial frequencies. The ferrets were trained to discriminate horizontal from vertical gratings. ***E***, Motion integration task stimuli: RDK consisting of black and white dots with varying coherence levels. RDK had to be discriminated based on their direction of motion (left or right). ***F***, Form integration task stimuli: linear Glass patterns with varying degrees of coherence. The ferrets were trained to discriminate horizontal from vertical patterns.

Following established methods, we tested behavioral and neural motion integration using random dot kinematograms (RDK; Fig. 1). These stimuli consist of randomly placed dots, each of which can either move in a global direction (signal dots), or in a randomly chosen alternate direction (noise dots). Integration over the signal dots results in the perception of a coherently moving pattern, the strength of which depends on the ratio of signal and noise dots (the so-called coherence level). Form integration was tested with a very similar stimulus, static Glass patterns (Glass, 1969; Glass and Pérez, 1973). These patterns are constructed from randomly distributed dot pairs (Fig. 1). For signal dot pairs, the axis connecting the two dots is oriented along a set orientation; noise pairs have a random orientation. Integration across signal pairs then reveals a global pattern, with a strength that is again determined by the coherence level.

We chose RDK and Glass patterns because they complement each other well: Processing of complex motion and form information has been associated with different visual streams in non-human primates and humans (Ungerleider and Pasternak, 2004). Yet, both stimulus types are constructed from the same elements, and can be used for quantitative measurements of integration thresholds in a highly comparable manner. In addition, both RDK and Glass patterns have been used to study the development of motion and form vision in monkeys and humans (for review, see Grinter et al., 2010; Kiorpes, 2016; Maurer and Lewis, 2018). Testing ferrets on RDK and Glass patterns therefore not only allows a comparison of their motion and form vision capabilities, but also provides a useful starting point for future developmental research.

Since very few visual behavioral experiments have been performed in ferrets (Doty et al., 1967; Pollard et al., 1967; Pontenagel and Schmidt, 1980; von Melchner et al., 2000; Hupfeld et al., 2006, 2007; Hollensteiner, 2015), testing their motion and form integration capacities required the development of appropriate behavioral paradigms. Here, we established a freely-moving paradigm and a head-fixed paradigm with greater control over viewing conditions. Using these paradigms, we found that ferrets show clear signs of motion and form integration. In general, ferrets were good subjects for visual psychophysics, performing tasks with low lapse rates, and behavior that could be well described by standard psychometric functions. Furthermore, we found behavioral limits of motion integration to be in close agreement with limits imposed by responses of PSS neurons, consistent with an involvement of higher-level visual areas in these tasks. In summary, our data establish the ferret as a viable animal model for studying more complex visual processes like motion and form integration.

## Materials and Methods

### Animals

All procedures were conducted in accordance with guidelines of the National institutes of Health and were approved by the Animal Care and Use Committee at Johns Hopkins University. A total of six adult female ferrets aged 5–50 months at the start of the experiments were used for the behavioral experiments. Each ferret participated in one to three studies of one to four months each (for the tasks each ferret participated in and their sequence, see Table 1). An additional three female ferrets and one male ferret aged 2–12 months were used for electrophysiology recordings. Our previous experiments have shown no difference in PSS motion integration in this age group (Lempel and Nielsen, 2019).

**Table 1.** Tasks that each ferret performed

Ferret	Acuity	Dots	Glass
F0	X	X	X
F4	X	X	
F2	X		
F6		X	X
F8		X	
F9		X	

Ferrets were first trained on the Acuity task, followed by the RDK task, followed by the Glass pattern task.

### Freely-moving behavior: design

#### Freely-moving setup

The behavioral box used for freely-moving behavioral tasks measured 100 cm long × 70 cm wide × 60 cm tall (Fig. 1*A*). The walls and the floor of the box were painted black, and the box did not have a ceiling. The behavior box was enclosed in its own room such that the ambient light and noise level could be controlled. A webcam (Logitech) affixed above the box was used to observe the ferret while performing the task. A cut-out within one of the short walls of the box accommodated a 24-inch VIEWPixx/3D monitor (VPixx) for displaying visual stimuli (refresh rate 120 Hz). Museum glass (True Vue) was placed directly in front of the monitor to protect the screen and minimize reflectance. All stimuli were generated using PsychToolbox (Brainard, 1997; Pelli, 1997; Kleiner et al., 2007), and stimulus timing and trial events were controlled by PLDAPS (Eastman and Huk, 2012). Two reward ports were positioned to the left and right side of the screen, and a third reward port was positioned in the center of the wall opposite the screen. Reward ports consisted of a metal spout used for reward delivery, surrounded by an infrared (IR) beam emitter and detector (Medical Associates Inc.). Breaks in the IR beam were used to detect port contacts. A fourth IR beam was installed to detect crossing the halfway point between the rear reward point and the monitor. Solenoid valves (Parker Hannifin) were used to dispense water reward.

#### General task design

All ferrets were trained on two-alternative forced-choice (2AFC) tasks. Preceding a given trial, a red LED was illuminated above the response port opposite the screen, indicating the start of a new trial (for task sequence, see Fig. 1*B*). Ferrets initiated a trial by activating this port. Trial initiation was rewarded with a small water reward (∼0.05 ml). A stimulus was presented on the monitor immediately after trial initiation, independent of which direction the ferret was facing at the time. With the exception of one RDK control experiment, all stimuli were shown on a gray background (50 cd/m^2^). Each stimulus was associated with one of the two response ports located on either side of the screen. If the ferret selected the correct port, a high tone (1-s duration) was played through the VIEWPixx speakers and a water reward (∼0.2 ml) was dispensed. If the ferret selected the incorrect port, a low tone (1-s duration) was played and no water was dispensed. After an incorrect choice, the ferret was required to activate the correct response port before it could initiate the next trial. Activation of the correct port was rewarded with a small amount of water (10–30% of the maximum possible reward size, dependent on the complexity of the task and stage of training). If ferrets demonstrated a significant bias toward one choice port, the amount of water reward dispensed at each port was varied by the experimenter until the ferret sampled both ports evenly.

In general, ferrets performed one session per day for 30–45 min, or ∼100 trials. We imposed no constraints on reaction time (i.e., the time between trial initiation and response port activation), other than ending trials during which the ferret failed to make a choice for a long time (around 120 s), which generally tended to only occur at the end of a testing session when motivation levels were low. Because it took the ferret some time to move between the two ends of the box, the shortest reaction times were around 2 s. In general, reaction time was ∼3 s. For all tasks, the stimulus presentation was tied to the choice behavior of the animal. More precisely, with the exception of the acuity task, in which the stimulus was removed when the ferrets reached the half-way point between both ends of the box, the stimulus remained visible until the ferret selected the correct port. In a subset of experiments, we measured both the time of the first response and the time of the correct response. On incorrect trials, the ferret took ∼2 s to correct its choice, meaning that the stimulus was presented for a total of ∼5 s.

#### Acuity task: stimuli, task design, training and animal inclusion criteria

Ferret acuity was assessed with an orientation discrimination task, using horizontal and vertical gratings of different spatial frequencies and contrasts (Fig. 1*C* & *D*). The contrast of each grating was varied from 0.05 to 0.95 Michelson contrast, and gratings were equiluminant with the background. To limit the effects of changing viewing distance on the spatial frequency of the stimulus, the stimulus was turned off once the ferrets crossed the IR beam at the midpoint of the box. Measured from this point in the box (50 cm from the screen), gratings had a diameter of 33°, and a spatial frequency between 0.09 and 0.36 cycles per degree (cpd). Horizontal and vertical gratings were paired with the left and right choice port, respectively. The orientation, spatial frequency, contrast, and phase of the grating were varied pseudorandomly across trials. Five ferrets (F0, F1, F2, F3, F4) were trained on this task. Two ferrets (F1, F3) failed to perform the task above 70% correct at a spatial frequency of 0.36 cpd and were excluded from further analysis.

#### Motion integration task: stimuli, task design, training and animal inclusion criteria

Ferrets were trained to discriminate between full-screen RDK with global translational motion to the left or right (Fig. 1*E*). RDK remained on the screen for the entire duration between trial initiation and triggering the correct response port. Stimulus metrics below are given from the last quarter of the box assuming that the ferrets made their decision after having turned around from the initiation port (viewing distance 75 cm). RDK measured ∼38° × 24°, and were composed of black and white dots (diameter 1.5°) with a density of 0.12 dots/°. Dot position and color were randomly initialized. Dots were randomly chosen to be signal or noise dots. The coherence parameter determined the percentage of signal dots on each trial, and ranged from 20% to 100%. Motion directions differed between signal and noise dots, but all dots moved at the same speed (also called “Brownian motion” noise; Pilly and Seitz, 2009; Schütz et al., 2010). More precisely, signal dots moved in the global stimulus direction (left or right), and noise dots in a direction randomly drawn from a uniform distribution of integers from 0° to 359°. On the first frame, each dot was assigned a randomly chosen lifetime that could range from 1 to 240 frames. Lifetime was decreased by one every frame. When the lifetime of a dot reached zero, the dot was probabilistically assigned to be a signal or noise dot according to the coherence parameter, and given a new lifetime of 240 frames. At this time point, noise dots were assigned new motion directions, drawn from the same uniform distribution as before. Dots that moved off-screen were re-plotted in a random position on the side of the screen opposite their direction of motion. This wrap-around was designed to maintain constant dot density. In addition, random repositioning after wrap-around and variable dot lifetime limited the subject’s ability to infer the direction of coherent motion from a single dot in isolation.

Before training on the full motion integration task, ferrets were trained to associate a 100% coherent stimulus moving toward the right with reward at the right port, and a leftwards moving stimulus with the left port. Once ferrets mastered this task (performance at or above 95% correct for at least two consecutive sessions), increasingly lower coherence levels were introduced systematically across days. Three ferrets (F0, F4, and F6) were trained in this manner, and all three successfully learned the task. After completion of training, psychometric motion integration functions were measured by varying coherence level and direction pseudorandomly from trial to trial. Dot speed was kept constant in each session. Different dot speeds were explored in different blocks of sessions (i.e., a new speed was tested after completion of tests with the previous one). Across blocks, speeds of 24°/s, 48°/s, and 72°/s (measured from the back of the box) were tested.

We ran a number of control experiments on individual ferrets (one ferret per experiment). In the first (ferret F4), we tested the impact of speed more directly by fixing the coherence at 60%, and varying dot speed pseudorandomly from 6°/s to 144°/s. In a second control experiment (ferret F0), we investigated the impact of dot lifetime by randomly setting maximum dot lifetime to either 240 or three frames on any given trial. In the third control experiment (ferret F6), we tested the influence of dot color by using only white dots on a black background.

#### Form integration task: stimuli, task design, training and animal inclusion criteria

Ferrets were trained to discriminate static linear Glass patterns based on their orientation (horizontal or vertical). As for the RDK task, stimuli remained on the screen for the entire trial duration, and stimulus parameters are given from a position in the last quarter of the box (viewing distance 75 cm). Stimuli covered the entire extent of the screen (38° × 24°). Each stimulus was composed of 200–250 white dot pairs (dot diameter 0.7°), with a distance of 1.2° between the dots in a pair. The position of all dot pairs was randomly initialized. At 100% coherence, all dot pairs were oriented either horizontally or vertically to create the global percept of a linear pattern (Fig. 1*F*). At lower coherences, dot pairs were randomly assigned to be signal or noise pairs, with the percentage of signal pairs determined by the coherence parameter. Noise pairs were assigned a random orientation drawn from a uniform distribution of integers from 0° to 359°. Ferrets began training on the task with fully coherent patterns until performance was at or above 85% for two consecutive sessions. At this point, increasingly lower coherence levels were gradually introduced. In the full task, coherence varied from 20% to 100%. Both the coherence level and the orientation of the Glass pattern varied pseudorandomly from trial to trial. Two ferrets (F0, F6) were trained on this task, and learned it successfully.

### Head-fixed behavior

#### Head-fixed setup

The head-fixed setup consisted of a headpost holder, body holder, and reward delivery system (Fig. 4*A*,*B*). The body holder was a custom-made plastic box with adjustable sides that ferrets could comfortably fit in, but that restricted their body movements (28.5 cm long, 10 cm wide, 10 cm tall; see Dobbins et al., 2007 for a similar design). The headpost holder allowed stabilization of the animal’s head by means of a headpost. This headpost was anchored in an acrylic cap attached to the skull with screws, which was implanted in an aseptic procedure under isoflurane anesthesia. The headpost holder height and position was custom to each ferret to ensure an ergonomic position.

The reward delivery system consisted of three spouts: two choice spouts, positioned 12 mm apart, and a neutral central spout that could be used for trial initiation (for a similar design for mice, see Marbach and Zador, 2017). The spouts were made from metal tubes (1 mm in diameter) bent into the correct shape. Licks were detected as changes in capacitance using an Arduino Uno board (Arduino) and the Arduino capacitive-sensing library. The spouts were electrically isolated from the rest of the setup. Each spout was mounted onto a pneumatic cylinder (McMaster-Carr), which used compressed air to retract and propel the spouts. This allowed each spout to assume two positions, one close to the animal, and one out of reach. The two choice spouts were additionally mounted on small translation stages (Newport) to control the distance between spouts. Finally, all spouts, along with the pneumatic cylinders, were mounted on a larger translation stage (Newport) to customize the distance of the spouts relative to the ferret. Solenoid valves (Parker Hannifin) were used to dispense water reward.

Stimuli were shown on the same VIEWPixx/3D monitor used in the freely-moving setup, which was placed 45 cm in front of the head-fixed setup. Stimuli again were generated using the PsychToolbox, and presentation was controlled using PLDAPS. We did not monitor eye movements during task performance for the data presented in this paper.

#### Task design for head-fixed paradigm

Ferrets were acclimated to the setup by receiving free water reward from each of the spouts while being head restrained. They were also permitted to freely move in and around the setup while the spouts were in motion to get comfortable with the noise of the gas pistons. This acclimation period lasted about 3 d. During this time, the position of the headpost holder and the spouts (relative to the ferret) were adjusted to optimize each animal’s position and access to the spouts.

Each training session was preceded by a short calibration phase (20 trials) during which the ferret was presented with a single choice spout at a time and no visual stimulus. This calibration period served to ensure that both choice spouts were treated equally by the ferret, which helped to lower response biases. How the ferret valued each spout was estimated by the lick frequency on that spout. The headpost position and the amount of water delivered on each spout were adjusted until the ferret demonstrated approximately equal lick frequency on each spout.

Two ferrets were trained on a head-fixed 2AFC task using RDK generated identically to the freely-moving paradigm. As before, RDK could move horizontally to the left or right, and ferrets had to respond to each motion direction by licking one of the two choice spouts. Each trial in the task began with an initiation phase (see later part of this section), followed by stimulus presentation (for task sequence, see Fig. 4*C*). Following a 200- to 300-ms delay, the two choice spouts were then moved close the animal, which had to lick one of them to indicate its choice. As for the freely-moving paradigm, no constraints were imposed on reaction time. In general, the first spout contact occurred after ∼650 ms from stimulus onset (i.e., within 350–450 ms from spout availability). If the ferret made the correct choice, the incorrect spout was retracted, and the ferret collected the full possible reward (∼ 0.15 ml) from the correct spout. Instead of delivering the entire amount of water at once, we divided it into a series of smaller rewards (∼30 µl per lick), and delivered the entire amount over a series of licks. The total amount of water per correct trial was controlled by limiting how long the spout was available to the ferret (1.5 s, or approximately five licks). We chose this reward strategy because ferrets tended to spill less of the smaller drops, allowing better control over reward amounts per trial. If the ferret selected the incorrect spout, it was immediately retracted, and the ferret had to correct its choice by licking the correct spout for a single drop of water (∼30 µl) before moving on to the next trial. A high tone was paired with correct choices, and a low tone was paired with incorrect choices. The stimulus remained on the screen until the ferret selected the correct spout (in general, it took ferrets an additional 1 s to correct an incorrect choice). If a ferret demonstrated a significant bias toward one response port, the amount of water reward dispensed at each port was varied by the experimenter until the ferret sampled both ports more evenly.

Trial initiation differed between the two ferrets. For F9, trials were passively initiated. After the intertrial interval (ITI), the stimulus appeared on the screen and the two choice spouts were presented. For F8, trials were actively initiated. In this case, a white square (3° × 3°) was presented in the center of the screen after the ITI, and the central spout was moved forward. When licks were detected on this spout, the trial began: The center spout was retracted, the stimulus appeared on the screen, and the two choice spouts were presented. Licks on the center spout were rewarded with a small amount of water (∼30 µl, divided into two to three licks performed during 0.5 s).

Before testing animals on the full motion discrimination task, ferrets were trained to associate 100% coherent RDK moving toward the right with reward at the right spout, and leftward RDK with reward at the left spout. Ferrets were introduced to this task by adding a fraction of instructive trials, in which only the correct spout was presented. This fraction was manually reduced both within and across sessions by the experimenter to maintain an overall minimum reward rate of 75–80%, which ensured a high level of motivation to perform the task. Once ferrets performed the task at or above 90% correct with no instructive trials, increasingly lower coherence levels were gradually introduced across days. The full task used coherence levels from 20% to 100%. For F9, trials at 100% coherence were doubly represented and instructed 50% of the time, which served to increase the overall reward rate and maintain a higher motivation level. Therefore, the number of uninstructed trials at 100% coherence was the same as the number of trials at every other coherence level, and the overall fraction of instructive trials was ∼14%. Instructed trials were excluded from further analysis. For F8, no trials at any coherence level were instructed, and all coherence levels were presented equally often. Coherence level and direction of motion varied pseudorandomly from trial to trial. Dot speed was fixed at 72°/s. The total number of trials per session that ferrets performed in the head-fixed setup was generally higher than the freely-moving behavior, but also more variable per ferret. In general (including initial training sessions at 100% coherence only), F8 would perform ∼200–500 trials, while F9 would perform ∼100–150 trials.

### Behavioral data: analysis and statistics

All data analysis was performed in MATLAB (The MathWorks). Data were concatenated across sessions for which the ferret performed at least 70 trials in the freely-moving paradigm and at least 100 trials in the head-fixed paradigm (for a summary of number of trials and sessions for each analysis, see Tables 2, 3). This criterion was imposed because behavioral training sessions in which ferrets performed fewer trials were indicative of decreased motivation and attention. Such training sessions were rare, and nearly always followed a break in behavioral training for a week or more. In one dataset for the freely-moving motion integration paradigm, a session in which the ferret performed 187 trials was excluded. Of the six sessions performed at the specified parameters for this task, the other five sessions had a mean of 100 trials with a SE of 7. We therefore felt that the session with 187 trials was an outlier that exerted undue influence on the fit of the psychometric function.

**Table 2.** Peak contrast sensitivity and maximum acuity estimates

Ferret	Peak contrast sensitivity (cpd)	Maximum acuity estimate (cpd)	Number of trials	Number of sessions
F0	0.17	0.70	1420	11
F2	0.17	0.60	1500	17
F4	0.20	0.65	1291	13
Mean	0.18 ± 0.01	0.65 ± 0.03	1404 ± 61	14 ± 2

Mean reported with SE.

**Table 3.** Threshold evaluations for each complex visual task

Task	Δ (50–68%)	Threshold at 75%	Threshold at 82%	Number of trials	Number of sessions
Dots, 72°/s					
F0	20.06%	35.62%	45.07%	1371	12
F4	14.05%	23.39%	32.17%	503	6
F6	12.79%	26.74%	34.98%	778	7
Mean, free	15.63 ± 2.24%	28.58 ± 2.65%	37.40 ± 3.92%	884 ± 256	8 ± 2
F9 (hf)	18.36%	30.55%	41.62%	568	4
F8 (hf)	28.35%	45.27%	59.48%	939	2
Mean, hf	23.36 ± 5.00%	37.91 ± 7.36%	50.55 ± 8.93%	753 ± 186	3± 1
Mean, all	18.72 ± 2.75%	32.31 ± 3.83%	42.66 ± 4.79%	832 ± 155	6 ± 2
Dots, 48°/s					
F0	18.93%	42.96%	49.98%	308	3
F4	15.01%	25.10%	35.56%	581	5
F6	18.91%	34.51%	47.76%	707	8
Mean	17.62 ± 1.30%	34.19 ± 5.16%	44.43 ±4.48%	532 ± 118	5 ± 1
Dots, 24°/s					
F0	19.89%	38.37%	50.89%	570	5
F4	17.67%	39.16%	47.37%	492	5
F6	16.67%	33.97%	44.20%	749	8
Mean	18.07 ± 0.95%	37.16 ± 1.61%	47.49 ± 1.93%	604 ± 76	6 ± 1
Glass					
F0	21.32%	44.31%	66.68%	766	6
F6	23.25%	48.94%	65.45%	616	5
Mean	22.28 ± 0.97%	46.65 ± 2.34%	66.06 ± 0.62%	691 ± 75	6 ± 1

Hf, head-fixed behavior; free, freely-moving behavior. Mean reported with SE.

We characterized behavior using three measures, a sided threshold Δ and thresholds corresponding to 75% and 82% correct. To compute Δ, we first computed signed contrast or signed coherence values, where negative values indicated conditions assigned to the left port, and positive values conditions assigned to the right port. We then determined the fraction of right port responses for each condition. The resulting psychometric curves were fit with a cumulative Gaussian using the Palamedes toolbox for MATLAB (Prins and Kingdom, 2018). Separate lapse rates were fit for left and right responses; 95% confidence intervals for psychometric curves were computed for a binomial using the Clopper–Pearson method (Clopper and Pearson, 1934). Finally, Δ was defined as the change in contrast or coherence required to increase the probability of a right choice from 50% to 68% of the maximum fraction of right responses (Busse et al., 2011). To compute thresholds based on percentage correct responses, we first averaged performance across the two sides. The resulting data were then fit with a cumulative Weibull function using the Palamedes toolbox, and thresholds determined as the contrast or coherence levels required to reach a performance of 75% or 82% correct.

Contrast sensitivity curves were fit using a double exponential of the form k_s_ (ω·k_ω_)^α^exp(-β·ω·k_ω_), where ω is the spatial frequency (Kiper and Kiorpes, 1994). The parameters k_ω_ and k_s_ capture shifts along the frequency and sensitivity axes, respectively, while α and β capture the steepness of the low and high frequency portions of the curve. This sensitivity curve was fit using an fminsearch algorithm in MATLAB. Error bars for sensitivity estimates were calculated by parametrically bootstrapping the data 1000 times, and fitting these bootstraps with psychometric functions to create a distribution of sensitivity estimates (Efron and Tibshirani, 1986). These represent 68% confidence intervals, or approximately one standard deviation from the mean.

The fidelity of the fit of a psychometric function was evaluated in two ways. First, the deviance of the fit was computed (Wichmann and Hill, 2001), which is defined as the extent to which the fit deviates from a saturated model in which there are no residual errors between the observed data and the fit. This tests how likely the fit is given the data. By convention, fits with deviance with significance <0.05 are rejected. The deviance and its significance were computed using the Palamedes goodness-of-fit function with 1000 simulations. Second, the mean squared error (MSE) between the model and the data were also computed.

To evaluate the statistical significance of differences between psychometric curves, likelihood ratios were computed of the form λ = (L(data | single curve)/L(data | independent curves)) (Hoel et al., 1971; Britten et al., 1992). –2ln(λ) is distributed as χ^2^ with degrees of freedom equal to the difference in dimensionality between the lesser and fuller model (Wilk’s theorem). Differences between two thresholds were tested for significance by parametrically bootstrapping the datasets 1000 times, and generating a distribution of thresholds for each. A Welch’s *t* test was then used to determine if the bootstrapped threshold distributions were significantly different. On the other hand, to test whether two thresholds were equivalent each dataset was bootstrapped 1000 times, and a distribution of the differences in the threshold values was generated. The equivalence bound was set to 2.5%, and the probability of threshold differences falling within this equivalence bound was derived from the difference distribution. This test for equivalence (TOST) yielded the p-value (Walker and Nowacki, 2011). Tests for equivalence were always performed on data from the same animal on the same task with two different stimulus conditions, and were only performed when the difference between psychometric curves was not statistically significant.

### Electrophysiology

#### Animal preparation and recordings

The electrophysiology experiments followed established methods detailed previously (Lempel and Nielsen, 2019). Briefly, experiments were performed in animals anesthetized with isoflurane (during surgical procedures: 2–3%, during recording: 0.5–1.5%). Animals were paralyzed with pancuronium (0.15 mg/kg/h) to prevent eye movements during recordings. A number of vital parameters (EtCO_2_, SPO_2_, heart rate, and EEG) were monitored continuously to maintain animals at appropriate anesthetic depths. Neural signals were detected using either custom-built tetrodes or 64-channel silicon microprobes (Masmanides lab, UCLA). Tetrodes were made using a 12-µm nichrome wire (California Fine Wire Company), and were plated using a gold solution (Sifco ASC) to reach final impedances of 150–500 kΩ. Silicon probes were gold-plated to reach final impedances of 150–300 kΩ. Signals were amplified and recorded using either a CerePlex Direct amplifier (Blackrock Microsystems) or a RHD2000 amplifier (Intan Technologies). Raw data were acquired at 30 kHz and filtered between 250 Hz and 5 kHz. A total of 10 penetrations were made across the three animals. The number of cells recorded concurrently (that were included in the analysis as significantly responsive and direction selective; see Data Analysis and Statistics) ranged between one and four cells. Of the 34 neurons included in the final analysis (see Data Analysis and Statistics), seven were recorded using the multichannel probes; the rest were recorded using tetrodes.

#### Stimuli

Visual stimuli were displayed on a gamma corrected 24” LCD monitor with a refresh rate of 120 Hz. The monitor was placed 25–35 cm in front of the ferret. RDK stimuli consisted of white dots shown on a black background. Otherwise, they were constructed identically to the behavioral experiments. Dot speed was fixed at 48°/s. For the tetrode recordings, we manually determined the preferred direction of the neuron under study in an initial experiment. The main experiment then consisted of RDK moving either in the neuron’s preferred direction or in the opposite (null) direction. For multichannel probe recordings, we initially determined the preferred direction of a selected neuron, and then used this direction and its opposite for the RDK. In addition to the initially selected neuron, other neurons that also strongly responded to the chosen RDK direction and that passed our selection criteria (see Data Analysis and Statistics) were also included in the analysis. In one multichannel recording experiment, we repeated the experiment with a different set of directions to drive a second group of cells with very different stimulus preferences, making sure that nonoverlapping sets of neurons resulted from these experiments. Coherences were varied from 10% to 100%, and each coherence level and direction were repeated 20 times in a pseudorandom sequence. A total of 20 blank trials were randomly interleaved throughout the experiment. Each RDK trial began with a static presentation of the first frame of the RDK for 2 s to control for responses to luminance changes. Dots then moved for 1 s, before presenting the last frame statically for 0.5 s. A blank black screen was shown between trials.

### Data analysis and statistics

Single unit isolation was performed off-line using custom MATLAB software. A spike detection threshold was set manually for each recording. Isolation was then based on multiple spike wave form characteristics (e.g., spike amplitude peak, area under the wave form, repolarization phase slope, etc.) recorded on the four tetrode channels or on neighboring channels of the silicon probe. Quality of isolation was confirmed by interspike interval (ISI) analysis. Units that displayed ISIs below 1.2 ms were excluded.

Identified single units had to pass two criteria to be included in further analyses. First, they had to be responsive, as indicated by a significant difference in responses to the preferred direction and blanks (Student’s *t* test, *p* < 0.01), as well as a response rate of at least 6 spikes/s for the best stimulus. Second, they had to be direction selective, quantified as a significant difference in responses between preferred and null direction (Student’s *t* test, *p* < 0.01). A total of 43 of 72 recorded neurons passed the responsiveness test, and 37 of the 43 neurons (86%) were considered direction selective.

We used established approaches to compute neurometric curves (Green and Swets, 1966; Britten et al., 1992). For a given single unit, the distributions of responses to its preferred and null direction were computed at each coherence level. Here, a response is defined as the number of spikes during the 1 s presentation of the moving RDK. A receiver operating characteristic (ROC) curve was then generated for each coherence level by setting a threshold, and determining the probabilities that the preferred or null direction elicited a response exceeding the threshold. Thresholds could range from 0 to each cell’s maximum response and were increased in steps of one impulse (similar to Britten et al., 1992). The area under the ROC curve (aROC) for a given coherence was taken to be a proxy for the fraction correct that would be obtained from listening to the neuron’s responses, and used to construct a neurometric function for each neuron. Finally, a cumulative Weibull function was fit to the estimated fraction correct at each coherence level using the Palamedes toolbox. Thresholds were estimated from each of these curves using a criterion of 75% correct. One neuron was excluded from further analysis because the Weibull function fit failed to converge, and two additional neurons were excluded because their aROC at 100% coherence was below 0.75. This resulted in a total of 34 neurons for the full analysis.

## Results

### Behavioral estimates of visual acuity

Most of the existing studies on ferret visual behavior tested the animals in setups in which they could move freely and earn food or water reward for performing a particular action (such as opening a door) in response to a visual stimulus (Doty et al., 1967; Pollard et al., 1967; Pontenagel and Schmidt, 1980; von Melchner et al., 2000; Hupfeld et al., 2006). These behavioral paradigms have the advantage of closely mimicking natural foraging behavior. They also do not require the cranial implants necessary for head-fixed paradigms. Thus, they lend themselves to expedient testing of large cohorts of animals, as may be required by some developmental studies. We therefore decided to first implement a similar freely-moving testing paradigm.

Our behavioral setup was designed for 2AFC discrimination tasks. It consisted of an open box with a screen placed on one wall for visual stimulus presentation (Fig. 1*A*). This screen was flanked by two choice ports, each consisting of an IR beam emitter and detector surrounding a water spout. Every visual stimulus was associated with one of the choice ports. A similarly constructed trial initiation port was placed in the middle of the wall opposite the screen. The animal initiated each trial by breaking the beam in this port (for task sequence, see Fig. 1*B*). This triggered stimulus presentation, and was rewarded with a small amount of water from the trial initiation port. The animal then had to respond to the stimulus by crossing the box, and selecting one of the two choice ports. Selection of the correct port resulted in the delivery of a water reward, removal of the stimulus (depending on the task), and ending of the trial. If the animal instead chose the incorrect port, it could not advance to the next trial before activating the correct port. Once the animal corrected its choice, a small water reward (1/10 of the full amount) was delivered to maintain engagement in the task. Requiring ferrets to choose the correct port to complete the trial was essential in encouraging an even sampling of both ports early in learning, and was useful in preventing biased behavior during all testing stages.

We first used this setup to estimate the ferret’s visual acuity behaviorally, a necessary prerequisite for determining appropriate stimulus parameters for the experiments to follow. We therefore trained ferrets to discriminate horizontal from vertical gratings (Fig. 1*C* & *D*), and varied grating contrast and spatial frequency across trials (contrast range: 0.05–0.95 Michelson contrast, spatial frequency range: 0.09–0.36 cpd). To limit changes in spatial frequency induced by changes in viewing distance, stimuli were only shown until the ferret crossed an IR beam halfway between the trial initiation port and the screen. The spatial frequency values given above are calculated from this point.

**Figure 2.**
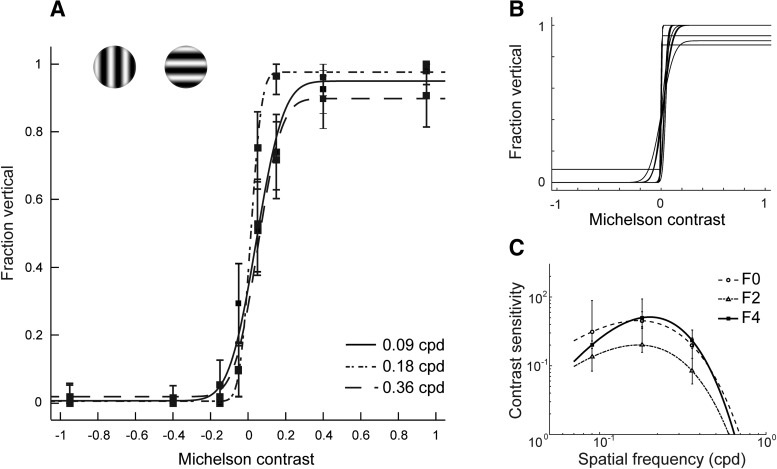
Behavioral estimates of visual acuity. ***A***, Psychometric curves for one ferret (F4) for three spatial frequencies. Error bars represent 95% confidence intervals (see Materials and Methods). ***B***, Psychometric curves fit to data from individual testing sessions using a spatial frequency of 0.18 cpd (ferret F4); 13 sessions are shown here. ***C***, Contrast sensitivity curves for each ferret. Error bars represent 68% confidence intervals (see Materials and Methods).

Visual acuity was determined for three adult ferrets (for animal selection criteria, see Materials and Methods). On average, the animals performed 103 trials per session (SEM: 4 trials) and achieved good performance for the easiest conditions (for training history for individual animals, trial and session numbers, and statistics per animal, see Tables 1–3). For the optimal spatial frequency (0.18 cpd) and the highest contrast, ferrets averaged 90% correct (SEM: 5.25% correct). In addition, their performance depended predictably on stimulus contrast and spatial frequency. This is clearly demonstrated by computing performance as a function of grating contrast for each spatial frequency (for an example ferret, see Fig. 2*A*): For all spatial frequencies, performance was close to chance level for low contrasts, but improved rapidly with increases in contrast. For further analysis, we captured the dependency of performance on contrast at each spatial frequency in the following way: we first computed performance as a function of grating contrast for each spatial frequency individually. To better account for potential response biases, performance was quantified as the fraction of vertical responses, and contrasts were expressed as “sided” contrasts, with –1 indicating a full contrast horizontal grating, and +1 a full contrast vertical grating. The resulting data were fit with a cumulative Gaussian to generate a psychometric function per spatial frequency. We then determined the sided contrast threshold Δ for each psychometric function, defined as the contrast increment required to go from 50% vertical responses to 68% of the maximum fraction of vertical responses (i.e., to 68% of 1 minus the lapse rate). Finally, the contrast sensitivity for a spatial frequency was defined as the inverse of Δ. The same approach has previously been used to evaluate acuity in mice (Busse et al., 2011).

In general, fits were based on aggregating behavior across multiple sessions for each ferret (*N* = 14 ± 2) to improve the estimation of contrast sensitivity. However, we verified in one ferret (F4) that performance was indeed consistent across days. For this animal, we fit data of individual sessions performed at a spatial frequency of 0.18 cpd, and determined a sided coherence threshold Δ for each session. These data confirmed that thresholds were highly similar across sessions (Fig. 2*B*, SD of Δ: 1.28%).

Finally, we used the collected data to compute a contrast sensitivity curve for each ferret. To this end, contrast sensitivity as a function of spatial frequency was fit with a double-exponential function (see Materials and Methods for details). The fit to the contrast sensitivity curve could then be used to determine two measures for every ferret: First, peak frequency, which corresponded to the spatial frequency with maximal contrast sensitivity. Second, cutoff frequency (also called maximum visual acuity), which corresponded to the spatial frequency with a contrast sensitivity of 1, and was determined by extrapolating the fit. Contrast sensitivity curves were highly similar across ferrets (Fig. 2*C*). They revealed an average peak spatial frequency of 0.18 cpd (SEM: 0.010 cpd), and average cutoff frequency of 0.65 cpd (SEM: 0.029 cpd).

Our behavioral estimates of contrast sensitivity and acuity are in good agreement with existing data on spatial frequency tuning in ferret area 17 (Baker et al., 1998). The optimal spatial frequencies for area 17 neurons range from ∼0.1 to 0.5 cpd, with a geometric mean of 0.25 cpd. Furthermore, neurons with the highest spatial frequency preference have an average bandwidth of 1 octave. Thus, not only is the behaviorally measured peak sensitivity close to the average optimal spatial frequency for area 17, the cutoff frequency also falls within the range of spatial frequencies that can elicit responses in area 17. While there are limitations to estimating acuity based on freely-moving behavior, the good match between behavior and neural data strongly supports our measurements. We therefore used these data as a basis for selecting stimulus parameters for the following experiments, in particular the size of dots in RDK and Glass patterns.

### Measurements of motion integration in freely-moving ferrets

Based on the success of the acuity experiment, we used the same freely-moving paradigm to probe higher-level motion processing by testing whether ferrets were capable of motion integration. RDK (Fig. 1*E*) have become the standard stimulus to assess visual motion integration performance, as they can be constructed so that integration across multiple dots is required for perceiving a global direction signal. Here, three ferrets were trained to discriminate RDK based on their global direction (left vs right) at a dot speed of 48°/s. Ferrets were introduced to this task at 100% coherence (i.e., with all dots moving in the global direction), and continued with 100% coherent motion until they performed at 80% correct or above. All ferrets demonstrated rapid learning, and reached criterion at 100% coherence within three to five sessions.

At full coherence, the direction discrimination task could theoretically be solved by attending to a single dot. Lower coherences require integration of motion information across dots, and therefore more accurately reflect motion integration capabilities. In addition, systematic changes in coherence levels allow threshold measurements, and thereby a quantitative assessment of motion integration capabilities. For this reason, we gradually introduced RDK with lower coherence once criterion performance was reached for the full coherence. After three to five additional sessions, ferrets performed the RDK task across a range of coherence levels (20–100% coherence). For the remaining analysis, only data from sessions in which the ferrets were assessed on the full range of coherences were included (3–12 sessions per animal).

**Figure 3.**
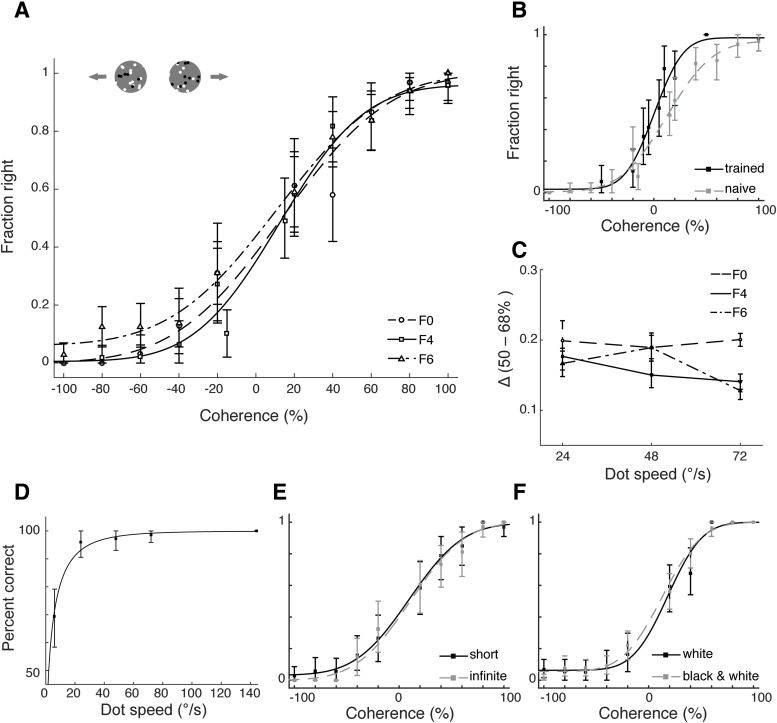
Motion integration thresholds. ***A***, Psychometric curves for each ferret on the motion integration task (dot speed 48°/s). ***B***, Impact of training on motion integration thresholds: performance for F4 at the time of initial threshold measurements, and after 11 additional sessions. ***C***, Sided coherence threshold Δ as a function of dot speed for each ferret. ***D***, Performance of ferret F4 for RDK of 60% coherence as a function of dot speed. ***E***, Performance comparison for short versus infinite dot lifetime (data for ferret F0). ***F***, Performance comparison for white dots on a black background versus black and white dots on a gray background (data for ferret F6). All error bars, with exception of C, represent 95% confidence intervals. Error bars for C are 68% confidence intervals.

In general, ferrets exhibited excellent performance on the easiest direction discrimination conditions: they performed on average at 98% correct for 100% coherent motion (SEM: 0.21%). These low lapse rates show that ferrets not only mastered the task, but that the behavior was under tight stimulus control. Consistent with a performance that is mainly driven by the information present in the motion stimulus, each ferret’s performance systematically depended on the coherence level. Once again, performance was quantified by computing a sided performance measure to appropriately address any response bias. More precisely, for each ferret we computed the fraction of right motion responses as a function of a sided coherence measure (–100%: full coherence, direction left; +100%: full coherence, direction right). These data were then fit with a cumulative Gaussian (Fig. 3*A*). Data from all ferrets could be fit well: No fits exhibited significant deviance from a saturated model (F0: deviance = 7.04, *p* = 0.143, F4: deviance = 10.02, *p* = 0.199, F6: deviance = 9.19, *p* = 0.128; see Materials and Methods), and on average, fits had a MSE of 2.34 (SEM: 0.078).

Motion integration capacity was then quantified based on the fits by computing a sided coherence threshold Δ (for data from individual ferrets and alternate threshold measures, see Table 3). Analogous to the computation of the contrast threshold, we defined Δ as the increase in coherence required to change the fraction of right responses from 50% to 68% of the maximum. This analysis yielded an average Δ across ferrets of 17.62% (SEM: 1.30%). Note, however, that these thresholds were determined after a limited number of sessions to quantify the general motion integration capacity of adult ferrets. Thresholds could be improved significantly by additional training (Fig. 3*B*): we continued to train F4 after the initial threshold measurement. A reevaluation after 11 additional sessions resulted in a sided coherence threshold Δ of 9.44%, significantly lower than the initial value of 15.01% (log-likelihood ratio test, –2*ln(λ) = 15.83, degrees of freedom = 2, *p* = 3.6576 × 10^−4^).

Performance on the RDK task might be expected to depend on dot speed. We therefore measured motion integration thresholds at three speeds: 24°/s, 48°/s, and 72°/s. No clear effect of speed on threshold was observed over this range (Fig. 3*C*). To investigate further, the performance of one ferret was evaluated at a fixed dot coherence (60%) and variable speed per trial (6–144°/s). In agreement with the larger dataset, the ferret’s performance in the control experiment was largely independent of speed (Fig. 3*D*). For speeds larger than 7.22°/s, the ferret performed above 75% correct, and performance was at or above 95% correct for speeds above 24°/s.

Finally, we performed two control experiments to test whether task performance was indeed due to integration of signals across dots, and not other stimulus factors. In the first control experiment, we tested the impact of dot lifetime, which determined how long each individual dot could remain a signal dot. Long lifetimes might allow a ferret to solve the task based on the trajectory of a single dot, rather than through integration. Thus, in the control experiment we randomly set lifetime to either 25 ms or 2 s at the beginning of every trial. Performance on the task (Fig. 3*E*) did not differ significantly between the two lifetime conditions (log-likelihood ratio test, –2*ln(λ) = 3.07, degrees of freedom = 2, *p* = 0.2156), and Δ values for each condition were equivalent within 2.5% of each other (TOST, *p* = 10^−3^), indicating that at least the ferret used in the control experiment was not following single dots to perform the task. In a second control experiment, we tested the impact of dot color. In the RDK experiments described so far, 50% of the dots were black and 50% white to provide some stimulus contrast to the animals (shown against a gray background). To rule out any effects of this color choice, we tested performance for RDK constructed from white dots only, shown on a black background (Fig. 3*F*). Again, the change in stimulus parameter did not affect performance in the control experiment (log-likelihood ratio test, –2*ln(λ) = 0.945, degrees of freedom = 2, *p* = 0.6233; Δ between conditions equivalent within 2.5%, TOST, *p* = 0). In summary, our experiments demonstrate a clear capacity for motion integration in ferrets.

### Tests of motion integration using a head-fixed paradigm

The freely-moving behavioral paradigm used so far excels in its ease of implementation. Ferrets generally learned quickly using this paradigm, and the results of the first two experiments confirm its suitability for psychophysical experiments. The main disadvantage of freely-moving behavior is a limited control over viewing distance and head position. Variable viewing distances and head positions complicate accurate estimates of how stimulus parameters such as stimulus speed, size, and spatial frequency influence task performance. Furthermore, freely-moving behavior does not lend itself as easily to simultaneous neural recordings, in particular using optical methods. For these reasons, we also developed a head-fixed behavioral paradigm. We then used this paradigm to measure motion integration performance in two additional ferrets, which allowed a direct comparison of behavior in the two paradigms.

**Figure 4.**
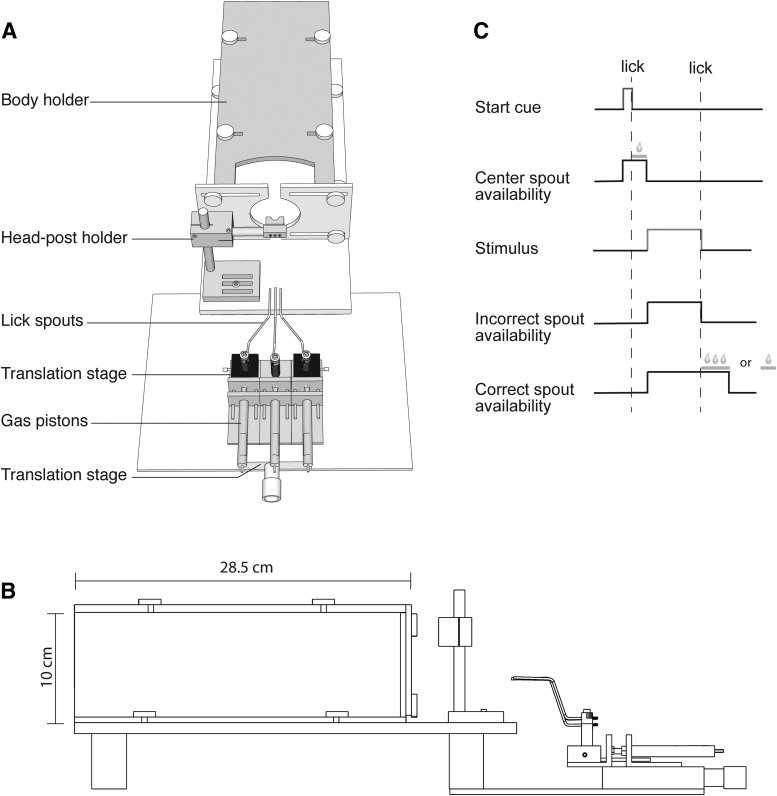
Head-fixed behavior paradigm. ***A***, Schematic drawing of the head-fixed behavior setup (top view). The setup consisted of three major components, a body holder, headpost holder, and the reward spouts. All three components could be moved relative to each other to allow the animal to assume a comfortable posture while in the setup, and to reach the spouts easily. Each spout could be moved independently between a retracted and a forward position by means of a gas piston. Animals could only lick the spouts when in the forward position. All spouts were mounted on a large translation stage to control their overall distance from the animal. In addition, the two peripheral spouts were mounted on two smaller translation stages to control the lateral distance between the spouts. This was necessary to make sure that animals could not activate more than one spout simultaneously. ***B***, Side-view of the head-fixed behavior setup. ***C***, Three-spout task design. A trial initiation cue was presented and the center spout was moved forward. When the ferret licked the center spout, a small reward was dispensed. Next, the center spout was retracted and stimulus presentation was triggered. The two choice spouts were moved forward. If the ferret licked the correct spout first, the incorrect spout was retracted, the stimulus removed, and the ferret received a large water reward. If the ferret contacted the incorrect spout first, it was also retracted. The ferret then had to contact the correct spout (which remained in position) to end the trial and receive a much smaller reward.

The head-fixed setup consisted of a headpost holder, a body holder, and three independently movable water spouts for reward delivery (Fig. 4*A*,*B*). Licks on the water spouts could be detected as changes in spout capacitance (see Materials and Methods for details). A screen for visual stimulus display was placed 45 cm in front of this setup. The headpost holder allowed fixation of the head by means of an implanted headpost, while the body holder limited movements by the rest of the body. A similar configuration has been used for auditory studies in ferrets (Fritz et al., 2003; Dobbins et al., 2007). At the beginning of training, the relative positioning of headpost holder and body holder were customized for each ferret to ensure a comfortable posture, and the animals were slowly acclimated to the setup (see Materials and Methods). The relative position of the water spouts was optimized such that the animal could lick them easily but distinctly: in other words, the animal could not contact more than one spout at the same time.

To mimic the structure of the freely moving behavioral paradigm, the central spout was designated as the trial initiation spout, while the two peripheral spouts were designated as choice spouts. Each spout had two positions: a retracted position, where the animal could not reach the spout, and a forward position, where the animal could lick the spout easily. Usually, ferrets did not lick while the spouts were retracted. Thus, the potential of motion artifacts throughout the trial, which could pose problems for combined behavioral and recording experiments, could be further limited by controlling the availability of the water spouts. A similar three-spout configuration, albeit with stationary spouts, has been used in mice (Marbach and Zador, 2017).

The head-fixed 2AFC task used the following design (Fig. 4*C*). During the ITI, all spouts were in the retracted position. After the ITI and a trial initiation phase (see next paragraph), a visual stimulus was presented on the screen. Following a brief delay, the two peripheral choice spouts were then moved forward, and the ferret had to respond to the visual stimulus by licking one of them. As in the freely-moving paradigm, we implemented a task design that forced sampling of both ports: In the case of a correct response, water was delivered as soon as the ferret contacted the spout. At the same time, the second spout was retracted. If the ferret instead chose the incorrect spout first, this spout was immediately retracted without reward. The ferret then had to correct its choice by contacting the remaining correct spout, rewarded with a much smaller amount of water, to end the trial. At this point, the correct spout was also retracted.

We explored two different trial initiation options with the two ferrets. In one ferret (F9), trials were passively initiated. For this animal, each trial began automatically after a fixed ITI by presenting a white square on the screen, which served to alert the animal to the upcoming stimulus presentation. In this design, the central spout was not used. In the other ferret (F8), we explored an active trial initiation. After the ITI elapsed, a white square again was presented on the screen. At the same time, the central spout was moved forward. The animal was required to lick this spout (rewarded with a small amount of water) to fully initiate the trial. The spout was then retracted, and the visual stimulus presented after a brief delay. While this full three-spout version of the task might be more challenging to learn, it offers the advantage of starting each trial with a central licking position, which could help reduce biases for the subsequent response choice.

**Figure 5.**
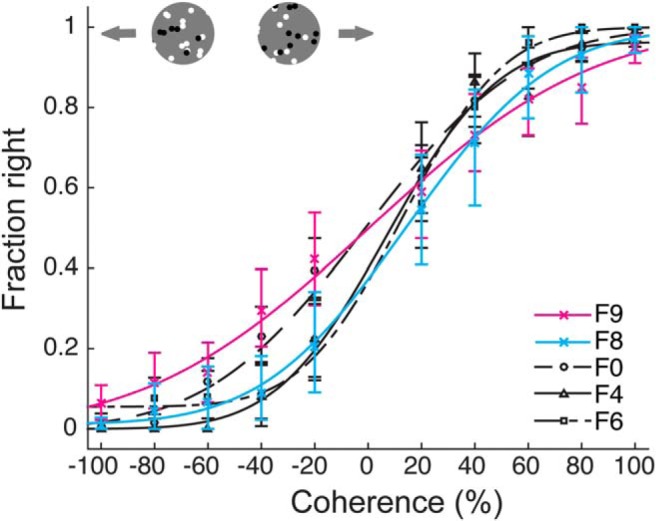
Comparison of motion integration thresholds measured using head-fixed and freely-moving paradigms. Psychometric curves from all ferrets for the motion integration task at 72°/s. Colored lines show the performance of the head-fixed animals, F8 and F9. Black lines show the performance of freely moving animals (F0, F4, and F6). Error bars represent 95% confidence intervals.

As before, ferrets were initially trained on the RDK direction discrimination using stimuli with 100% coherence (dot speed 72°/s). Both ferrets learned the task, and participated well. F9 performed at 80% correct or above within one week of training, while F8 reached the same criterion within three weeks of training. F9 performed 142 trials per session on average (SEM: 14 trials), and F8 performed 469 trials on average (SEM: 15 trials). The data collected from the head-fixed task exhibited many of the same properties observed for the freely-moving paradigm (Fig. 5). First, ferrets again performed the task with low lapse rates (3.81 ± 1.96%, mean and SEM), comparable to the lapse rates for freely-moving animals (for 72°/s: 0.91 ± 0.14%, mean and SEM). Thus, despite the fact that the different setups might have been expected to produce differences in motivational state (such as the overall willingness to perform the task, the subjective cost incurred by an error, etc.), performance was under similarly strong stimulus control in both paradigms. Second, psychometric functions were again well described by a cumulative Gaussian (F9: deviance = 1.76, *p* = 0.802, MSE = 0.25, F8: deviance = 3.69, *p* = 0.760, MSE = 0.67). Sided coherence thresholds Δ based on these fits were 18.36% for F9 and 28.35% for F8. This places F9’s performance well within the range of Δ values observed for the same speed in the freely-moving paradigm (12.79% - 20.06%), while F8’s performance was somewhat worse. Note, however, that since F8 performed so many trials per session, we used only two sessions to compute Δ for this ferret. For all other ferrets, 4–12 sessions were used to determine the threshold. It is possible that extra training provided by additional sessions would have lowered thresholds for F8 to be more similar to the other ferrets.

The speed of task learning, low lapse rates, and fidelity of psychometric function fits demonstrate that a head-fixed 2AFC task design can be used for visual psychophysics in ferrets, thus opening the door for future work combining neural recordings with visual tasks in ferrets. Since no major differences were observed between the three- and two-spout versions of the task, both are viable designs. Moreover, the general agreement of thresholds across paradigms suggests that they may be used to complement one another.

### Form integration capacity of adult ferrets

The experiments described above demonstrate clearly that ferrets are capable of complex motion vision. Another important aspect of higher-level vision, at least in primates, is the ability to process form information, a function that is usually associated with different visual areas than processing of motion information (Ungerleider and Pasternak, 2004). While it is unclear whether the same holds for ferrets, at least their basic capacity to perform tasks requiring general form discrimination has been demonstrated (Doty et al., 1967; Pontenagel and Schmidt, 1980). Rather than investigating the most complex aspects of form vision (such as object recognition), we decided to study form vision at a comparable level of complexity to the RDK motion integration task. To this end, we chose Glass patterns (Glass, 1969; Glass and Pérez, 1973). In addition to consisting of similar elements as the RDK, Glass patterns offer the advantage of allowing measurements of form integration thresholds in a comparable manner to the motion integration thresholds. For these reasons, they have been used to assess the development of sensitivity to global form sensitivity in humans and monkeys, and to compare it to the development of sensitivity to global motion (Lewis et al., 2004; Kiorpes et al., 2012). Glass patterns can be constructed to yield different global patterns, including concentric, radial or linear forms. Here, we chose linear Glass patterns (Fig. 1*F*), because they allowed us to continue to use a 2AFC task very similar to the task used for the RDK.

**Figure 6.**
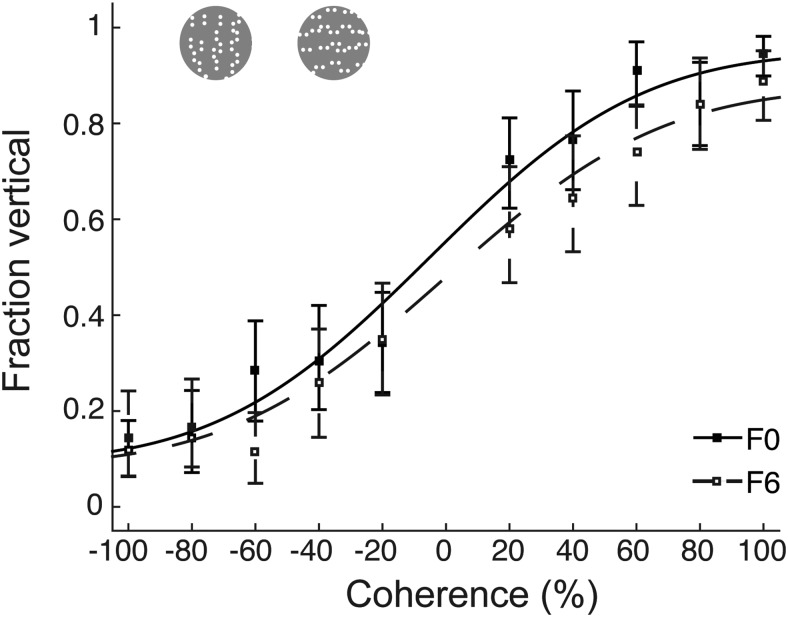
Form integration thresholds measured using Glass patterns. Psychometric curves for Glass pattern stimuli for two ferrets. Error bars represent 95% confidence intervals.

Two ferrets were trained to discriminate horizontal from vertical Glass patterns. All tests used the freely-moving paradigm because of its easier implementation. As for the RDK, Glass patterns were introduced at 100% coherence, and remained at this level until ferrets achieved a criterion performance of 80% correct. Lower coherence levels were then gradually introduced in subsequent sessions. In the following analyses, data were limited to behavioral sessions that used the full range of coherences (20–100%). Ferrets were able to learn the basic Glass pattern task (Fig. 6), and achieved good performance on the easiest condition (F0: lapse rate = 91%, F6: lapse rate = 87%). Their overall behavioral data were once again well described by cumulative Gaussian functions (F0: deviance = 0.379, *p* = 0.774, MSE = 2.06; F6: deviance = 0.401, *p* = 0.5780, MSE = 1.31). Sided form coherence thresholds Δ were computed identically to the sided motion coherence thresholds to facilitate a comparison across tasks (for other thresholds, see Table 3). For F0, this analysis resulted in a threshold of Δ = 21.32%; the threshold for F6 was Δ = 23.25%. Both ferrets were previously tested on the freely-moving motion integration task, allowing a direct comparison of thresholds between the two tasks. For both ferrets, thresholds were significantly higher in the Glass pattern than the RDK task (F0: 2.38% difference in Δ between RDK at 48°/s and Glass pattern task, *p* = 7.919e-189, *t* = 32.77, df = 1998; F6: 4.34% difference in Δ, *p* = 7.169e-138, *t* = 27.08, df = 1998). This suggests that although they were able to learn both tasks, the ferrets found the Glass pattern task more challenging than the RDK task. The increased difficulty may reflect genuine differences in processing of form versus motion information in ferrets. However, since both ferrets were trained on the motion before the form task, we cannot rule out that the training sequence caused interference between the two tasks.

### Comparison of behavioral and neural motion integration limits

**Figure 7.**
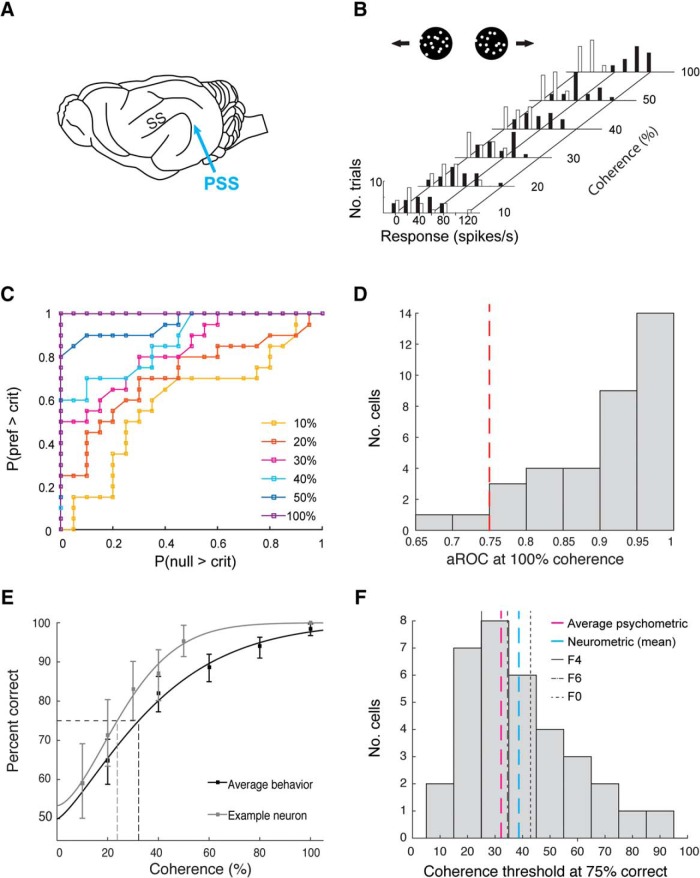
Comparison of neurometric and psychometric motion integration thresholds. ***A***, Sagittal view of the ferret brain, with the suprasylvian sulcus (SS) and PSS indicated. ***B***, Firing rate distributions for an example PSS neuron, evoked by RDK of different coherence levels moving in the neurons preferred direction (black bars) or its null direction (white bars). Each bar indicates the number of trials on which a neuron exhibited a particular firing rate. ***C***, ROC curves generated from the distributions in ***B***. ***D***, aROC values for all directionally selective and significantly responsive neurons (*N* = 36) at 100% coherence. Red dashed line at 0.75 indicates criterion cutoff. ***E***, Comparison of an example neurometric function, computed for the neuron shown in ***C***, ***D***, to the average psychometric function. The average psychometric function was generated by fitting behavioral data collapsed across all three ferrets tested in the freely-moving paradigm. The threshold for the average psychometric function, using a criterion of 75% correct responses, is also indicated. Error bars indicate 95% confidence intervals. ***F***, Distribution of 75% correct coherence thresholds across all directionally selective, significantly responsive neurons with aROC values of 0.75 or above at 100% coherence (*N* = 34). Also shown are the mean of this distribution, the threshold based on the average psychometric function (see ***E***), and the thresholds of each of the three ferrets, all using the same criterion of 75% correct.

The experiments described above were designed to test behaviorally whether ferrets are able to integrate motion and form information, functions that are associated with mid-level visual areas such as MT and V4 in the primate (Ungerleider and Pasternak, 2004; Orban, 2008). An important aspect of establishing higher-level vision research in ferrets will be to identify the areas supporting the more complex visual behavior we observed. Little is currently known about processing of form information in ferret visual cortex outside of area 17 and 18. The same largely holds for motion processing. However, previous studies have identified one higher visual area involved in motion processing (Philipp et al., 2006; Hupfeld et al., 2007). This area, called PSS or PMLS, is located in the posterior bank of the suprasylvian sulcus (Fig. 7*A*). Building on this finding, we recently demonstrated that PSS shows the same signatures of complex motion processing that are observed in primate MT (Lempel and Nielsen, 2019). This includes a significant change in the degree of motion integration between area 17 and PSS: As in the primate motion pathway (Born and Bradley, 2005; Orban, 2008), motion processing in area 17 is concerned with local motion signals, while PSS extracts integrated, global motion signals.

This provides the opportunity to test whether the behaviorally observed motion integration is consistent with limits imposed by neural activity in higher-level area PSS. Ultimately, the contribution of PSS to visual motion integration behavior will need to be addressed by recording and manipulating neural activity during the task. However, as a first step, we compared behavioral thresholds with thresholds of PSS neurons recorded in a different group of animals during anesthetized experiments. In these experiments, we used tetrodes or multi-site silicon probes to isolate responses of individual PSS neurons. For each neuron, we first determined the preferred direction. We then collected responses to repeated presentations of RDK with varying coherence levels. RDK could move either in the neuron’s preferred direction, or the opposite (null) direction at a fixed speed of 48°/s. These data allowed us to use a signal detection theory approach to determine the likelihood of correctly detecting the preferred direction based on the firing rates of the recorded neuron (Green and Swets, 1966). More precisely, the measured firing rate distributions for preferred and null direction were used to calculate ROC curves at each coherence (Fig. 7*B*,*C*). The probability of correctly detecting motion in the preferred direction at a coherence level could then be estimated from the area under the corresponding ROC curve (aROC). aROC values need to reach reasonably high levels for the remainder of the analysis to be meaningful. Figure 7*D* therefore shows aROC values at 100% coherence for 36 responsive and directionally selective neurons. To be included in further analysis, neurons were required to reach a minimum aROC value of 0.75. A total of 34 neurons remained after this step, and were each fit with a Weibull function to capture the dependency of detection probability on coherence level (Fig. 7*E*). This neurometric function was used to estimate the integration threshold for each neuron as the coherence required to reach 75% detection probability. A similar approach has been used previously to compare neural responses in primate MT to behavioral motion integration performance (Britten et al., 1992).

Figure 7*F* plots the resulting distribution of PSS neurometric thresholds and the matching behavioral thresholds collected from three ferrets tested on the same dot speed in the freely-moving paradigm. To directly compare neural and behavioral results, we recomputed behavioral thresholds as the coherence levels required to reach 75% correct, instead of the sided coherence threshold used earlier (Table 3). We also collapsed the behavioral data across animals to generate an average psychometric curve, and computed its threshold. Despite the fact that neurometric and psychometric thresholds were derived in different groups of animals, the two datasets were in close agreement (Fig. 7*F*). The psychophysical threshold of each individual ferret fell within the interquartile range of the neurometric distribution (26.47th, 50.00th, and 67.65th percentiles of the distribution, respectively), and the estimated threshold of the aggregate ferret behavior data was very close to the median of the neural distribution (47th percentile, average psychometric function threshold: 32.22% coherence, median neural threshold: 34.63%). Thus, neural limits on motion integration imposed by PSS are in close agreement with the behaviorally observed limits. More work is necessary to fully establish the role of PSS and other visual areas in complex motion processing, and to identify the areas involved in processing of Glass patterns in ferrets. However, our findings represent a promising sign that at least the RDK task taps into functions supported by the ferret’s higher-level visual areas.

## Discussion

To date, there have been few detailed behavioral studies on the visual capabilities of ferrets. Here, we systematically tested their ability to discriminate simple gratings and more complex stimuli requiring integration. The first important conclusion derived from our experiments is that ferrets are good subjects for visual psychophysics. For all stimulus types tested, their performance systematically depended on critical stimulus parameters such as spatial frequency or coherence level, in a way that was well captured by standard psychometric functions. In addition, performance was reliable across days, and ferrets usually performed a reasonable number of trials per day. Ferrets consistently were able to perform tasks with low lapse rates. This is important, as it confirms that behavior is tightly controlled by the stimulus. It is worth noting that to reliably achieve these low lapse rates, attention needed to be paid to any response bias exhibited during training and testing. Individual animals at times developed a preference for one of the two response ports, and chose it regardless of the stimulus. Requiring the animals to end each trial by choosing the correct spout helped to significantly reduce the occurrence of response biases. For the head-fixed behavior, it was additionally important to ensure that all spouts were equally reachable by the animal. Any remaining biases could then be eliminated by temporarily changing the reward ratio of the response spouts.

So far, head-fixed paradigms have not been used to investigate visual behavior in ferrets. Here, we demonstrate that head-fixed and freely-moving behavior can be used equally well for vision research in this species. The two paradigms have different advantages and disadvantages: While the freely-moving paradigm lacks complete control over certain stimulus parameters (including viewing distance), it mimics natural behavior, requires no cranial implants, and animals usually take to it quickly. It thus lends itself to studies requiring screening of larger cohorts of animals. The head-fixed paradigm, on the other hand, requires implants and takes longer to train, but provides complete control over viewing conditions. Because of the fixed head position, it also lends itself more easily to combined recording and behavior experiments, in particular if neural activity is to be recorded optically (e.g., using two-photon calcium imaging).

In the experiments described here, we controlled the animal’s head position and distance relative to the screen, but did not attempt to monitor eye position. Using the head-fixed setup to record neural activity in visual cortex while animals perform a task will require that the stimulus can be maintained at a stable position relative to center of gaze, to ensure that stimuli remain within the receptive fields of the neurons under study. Tracking of eye position is feasible in ferrets and could be added to the setup (Stitt et al., 2018). This, at the minimum, would allow monitoring of eye position during an experiment, which could be used to eliminate trials contaminated by saccades or large deviations in eye position from a desired location. Monitoring of eye position might also enable tasks in which ferrets are trained to fixate a target, which would more tightly control stimulus position. Fixation tasks likely will also be required to be able to present stimuli peripherally. Whether ferrets can indeed be trained to fixate a stimulus remains an open question to be investigated in future experiments. In any case, the addition of eye tracking to the setup will require the development of efficient strategies to calibrate the eye tracking signal, either by developing tasks that require ferrets to fixate targets presented at different positions, or through other automated procedures like the ones developed for eye tracking in rats (Zoccolan et al., 2010).

The second important conclusion derived from our experiments is that ferrets show clear behavioral evidence of higher-level visual processing, as indicated by their ability to perform motion and form tasks that require integration of information across multiple elements. The motion integration thresholds measured here are consistent with the results of a previous ferret study, in which animals were tested on their ability to discriminate coherent from random motion (Hupfeld et al., 2006). In this task, ferrets achieved a performance level of 75% correct for coherence levels of around 20%. These thresholds are lower than the thresholds of 30–37% determined here, most likely because of the simpler discrimination task. The studies also differ in other parameters such as the lapse rates and the number of trials performed per day, which might have affected the measured psychometric functions. Motion integration thresholds have also been measured in the cat, a carnivore like the ferret, using stimuli very similar to the ones employed here. Across two studies, motion integration thresholds for cats (measured either at 70% or 75% correct performance) ranged from 5% to 15% (Rudolph and Pasternak, 1996; Mitchell et al., 2009). These thresholds are lower than the ones measured for ferrets in this study, which could indicate that cats are better able to integrate motion signals, but might also be due to differences in the amount of training animals received in the different studies.

Ferrets were similarly able to perform a form integration task. Note that we chose to use linear rather than concentric Glass patterns here to more closely match motion and form tasks. In human subjects, thresholds for linear Glass patterns differ from those for more complex patterns containing curvature, such as concentric Glass patterns (Wilson and Wilkinson, 1998; Wilson et al., 1997). It has been proposed that this difference arises because Glass patterns containing curvature tap into the curvature tuning in higher-level areas like V4 (Gallant et al., 1993; Pasupathy and Connor, 1999, 2001). Yet, these conclusions might be confounded by effects of viewing Glass patterns through circular apertures, as is commonly the case (Dakin and Bex, 2002). Nonetheless, probing form integration in ferrets with curved Glass patterns remains an interesting topic for future investigations.

Generally, processing of RDK and Glass patterns has been associated with higher-level visual areas. In monkeys, MT in particular has been considered central for the processing of RDK (Newsome and Pare, 1988; Salzman et al., 1990; Britten et al., 1992). One important reason for this conclusion is the close agreement between neurometric functions of MT neurons and behaviorally measured psychometric functions (Britten et al., 1992; Shadlen et al., 1996). In contrast to the primate studies, we so far have not recorded neurons and behavior simultaneously in ferrets doing motion or form integration tasks. Yet, we have recently shown that PSS is a higher-level motion area exhibiting similar degrees of motion integration as MT (Lempel and Nielsen, 2019). These findings, combined with the close agreement of psychometric and PSS neurometric thresholds observed here, support the notion that performance on the RDK task indeed depends on higher-level visual areas in ferrets as in primates. This would also be consistent with the observation that PSS lesions impact ferrets on a motion detection task using RDK (Hupfeld et al., 2007). Comparisons to the cat further support this argument, as lesions of cat suprasylvian sulcus, a region containing motion areas that are likely closely related to ferret PSS (Philipp et al., 2006), similarly disrupt motion integration thresholds (Rudolph and Pasternak, 1996). Ferrets ability to perform the form integration task then raises the intriguing possibility that there is a matching higher-level visual area for form processing, to be located in future experiments.

Finally, it should be noted that testing ferrets on RDK and Glass patterns required certain adjustments, in particular an increase of the dot sizes, relative to experiments in humans and non-human primates to accommodate their poorer visual acuity. Our own experiments estimated peak contrast sensitivity to fall around 0.18 cpd, and a maximum acuity of 0.65 cpd. As discussed above, these behavioral acuity estimates are consistent with spatial frequency tuning curves of area 17 neurons in ferrets (Baker et al., 1998). They also agree with an earlier behavioral study testing the ability of ferrets to detect gratings of different spatial frequencies and contrasts (von Melchner et al., 2000).

In conclusion, our experiments firmly establish the feasibility of visual psychophysics in ferrets, including on experiments thought to tap into higher-level visual functions. RDK and Glass patterns have been used previously to study the development of motion and form vision pathways in monkeys and humans (Grinter et al., 2010; Kiorpes et al., 2012; Maurer and Lewis, 2018). Our findings open the door to perform similar experiments in ferrets. Because of their early birth (Sharma and Sur, 2014), and the ability to systematically alter visual experience during development (Chapman and Stryker, 1993; Chapman and Gödecke, 2000; White et al., 2001; Li et al., 2006; Van Hooser et al., 2012), this presents exciting opportunities for future developmental research.

## Synthesis

Reviewing Editor: Tatiana Pasternak, University of Rochester Decisions are customarily a result of the Reviewing Editor and the peer reviewers coming together and discussing their recommendations until a consensus is reached. When revisions are invited, a fact-based synthesis statement explaining their decision and outlining what is needed to prepare a revision will be listed below. The following reviewer(s) agreed to reveal their identity: Leo Lui, Jennifer Bizley. Note: If this manuscript was transferred from JNeurosci and a decision was made to accept the manuscript without peer review, a brief statement to this effect will instead be what is listed below.

The reviewers were largely satisfied with the revision. Reviewer 1 suggested two minor changes: adding a statement in the Methods about eye movements not being recorded and a clarification to the legend of Fig 7f. Please address those.

An additional comment from the Reviewing Editor concerns potential relevance of the work that not cited in the manuscript, which reported the effects of PMLS/PLLS lesions on motion integration thresholds in a cat(Rudolph and Pasternak, 1996). Because of the similarity between cat and ferret visual systems, their motion thresholds and the likely correspondence between PMLS/PLLS in the cat and PSS in the ferret, the authors may find this paper useful, particularly when evaluating motion coherence thresholds measured in their animals. Here, the interspecies comparison would be more relevant than the comparison with monkey thresholds included in the initially submitted version.

REVIEWER 1.

Comments to the authors

The authors have addressed all the points that were identified from a previous submission. The changes that were made to the paper have addressed the weaknesses.

2 minor points.

The authors may like to state that eye movements were not recorded in the methods. Saves readers getting to the end of the paper to work this out.

In the new Figure Legend 7f, "all neurons" is confusing. Maybe neurons that crossed the threshold or something to that effect.

REVIEWER 2

Comments to the Authors:

The text additions and edits add to the clarity of the manuscript. The authors have done an excellent job of responding to feedback on a previous version of this manuscript.
